# ECM Decorated Electrospun Nanofiber for Improving Bone Tissue Regeneration

**DOI:** 10.3390/polym10030272

**Published:** 2018-03-06

**Authors:** Yong Fu, Lili Liu, Ruoyu Cheng, Wenguo Cui

**Affiliations:** 1 Department of ENT and Head & Neck Surgery, The Children’s Hospital Zhejiang University School of Medicine, 3333 Bingsheng Road, Hangzhou 310051, China; 2 Orthopedic Institute, Soochow University, 708 Remin Road, Suzhou 215006, China; 13295152709@163.com (L.L.); cry214657@126.com (R.C.); 3 Shanghai Institute of Traumatology and Orthopaedics, Shanghai Key Laboratory for Prevention and Treatment of Bone and Joint Diseases, Ruijin Hospital, Shanghai Jiao Tong University School of Medicine, 197 Ruijin 2nd Road, Shanghai 200025, China

**Keywords:** elctrospun nanofibers, extracellular matrix, biologically active, osteogenic differentiation, scaffolds

## Abstract

Optimization of nanofiber surface properties can lead to enhanced tissue regeneration outcomes in the context of bone tissue engineering. Herein, we developed a facile strategy to decorate elctrospun nanofibers using extracellular matrix (ECM) in order to improve their performance for bone tissue engineering. Electrospun PLLA nanofibers (PLLA NF) were seeded with MC3T3-E1 cells and allowed to grow for two weeks in order to harvest a layer of ECM on nanofiber surface. After decellularization, we found that ECM was successfully preserved on nanofiber surface while maintaining the nanostructure of electrospun fibers. ECM decorated on PLLA NF is biologically active, as evidenced by its ability to enhance mouse bone marrow stromal cells (mBMSCs) adhesion, support cell proliferation and promote early stage osteogenic differentiation of mBMSCs. Compared to PLLA NF without ECM, mBMSCs grown on ECM/PLLA NF exhibited a healthier morphology, faster proliferation profile, and more robust osteogenic differentiation. Therefore, our study suggests that ECM decoration on electrospun nanofibers could serve as an efficient approach to improving their performance for bone tissue engineering.

## 1. Introduction

Large-sized bone defects cannot heal by themselves, due to the limited regenerative capability of bone tissue; therefore, a variety of bone regeneration strategies have been developed in the past 30 years to address this critical clinical problem [1,2,3]. Among these approaches, bone tissue engineering, which uses a combination of scaffolds, cells, and growth factors to regenerate new bone, still holds the promise of fully solving this important matter [4,5]. As one of three key components for bone tissue engineering, scaffolds play a pivotal role in successfully regenerating bone. Researchers have developed various approaches to fabricating scaffolds for bone regeneration, such as salt-leaching, freeze-drying, gas foaming, phase separation, and sintering, etc. [6,7,8,9,10,11]. For instance, Ma et al. developed a series of scaffolds with controllable pore size, porosity and other parameters using a phase separation method [6]. Bone tissue is generally considered to be a nanomaterial with nanoscale collagen fibers and hydroxyapatite organized into a hybrid structure [12,13]. However, most of these methods are capable of making scaffolds with micrometer features and fail to mimic the natural structure of bone.

Electrospinning is a facile and versatile method for fabricating fibrous scaffolds from a rich variety of materials [14,15,16]. Electrospun scaffolds possess very high surface-to-volume ratio, pore sizes ranging from several to tens of micrometers, and adjustable porosities of up to more than 90% [17]. Therefore, electrospun fibrous scaffolds have been successfully applied in neural, skin, cardiovascular, heart and bone tissue engineering [18,19,20,21]. In in the context of bone tissue engineering, electrospinning can be easily leveraged to make nanofibers similar to those of collagen fiber in bones, thus precisely emulating the natural structure of bone nanostructure [22,23,24]. Poly (l-lactic acid) (PLLA) is an FDA-approved polymer that possesses excellent biocompatibility and biodegradability; therefore, it has been extensively used for bone repair [25,26]. For instance, PLLA has been successfully electrospun into nanofibers to mimic the nanostructure of collagen fibers in natural bone and was able to promote bone formation [27]. One of the advantages of using nanofibrous materials is that they can effectively interact with stem cells and stimulate autocrine/paracrine growth factor signaling pathways [28]. A study led by Laurencin showed that delivery of rat MSC along with biomimetic electrospun matrices enhanced ligament repair by showing increased mechanical strength and improved tissue organization [29]. However, structural similarity does not provide all properties needed for bone regeneration. For instance, the surface properties of the electrospun nanofibers also play a crucial role in their performance once implanted in a bony environment [30]. Unfortunately, the surface properties of most electrospun nanofibers cannot meet the requirement for optimal bone regenerative performance. These materials are usually made of natural or synthetic polymers, which are not capable of directing cellular differentiation down to the bone lineage [19,31]. Therefore, a surface modification strategy combined with electrospun nanofiber scaffolds is highly desirable in bone tissue engineering.

Herein, we report an extracellular matrix (ECM) based surface modification approach for electrospun nanofibers to substantially improve their performance during bone repair and regeneration. ECM is composed of structural and functional molecules secreted by the resident cells of each tissue; therefore, ECMs are tissue specific, suggesting ECM secreted by cells from different tissues might have unique functions [32,33,34]. Due to these desirable properties of ECM, various medical devices, such as wound dressings and hernia patches, have been developed and have demonstrated satisfactory clinical outcomes [35]. Thus, we hypothesized that by modifying the material surface with a specific type of ECM, the cell behavior on the surface may be tailored by controlling the presentation of ECM deposition. In this study, in order to enhance the osteogenic differentiation of osteoblast/osteoprogenitor cells on electrospun nanofiber, we used MC3T3-E1 cells as a tool to deposit osteogenic ECM on electrospun PLLA surface by culturing the cells on PLLA nanofiber followed by a decellularization process to remove the cellular components. ECM decoration on PLLA nanofiber significantly enhanced cell attachment, as well as cell growth and osteogenic differentiation of mouse bone marrow stromal cells (mBMSCs). This work should set up an example of using ECM as a surface modification approach to obtain desirable biological properties in electrospun materials.

## 2. Experimental

### 2.1. Electrospinning

To prepare a PLLA nanofibrous scaffold, PLLA (*M*_n_ = 60.145 kDa, MWD = 1.64) pellets were dissolved in dichloromethane (DCM)/*N*,*N*-dimethylformamide (DMF) (50/50 vol %) solvent system at 12 wt %. The PLLA solution was fed into a plastic syringe connected to a stainless steel capillary tube at a feeding rate of 2.0 mL/h, controlled by a micro infusion pump (WZ-50C2, Zhejiang University Medical Instrument Co., Hangzhou, China). A positive DC high-voltage power supply (DW-P303-1AC, Tianjin Dongwen High-Voltage Power Supply Factory, Tianjin, China) was connected to the capillary tube to generate a high electric field of 2.0 Kv/cm between the capillary tube and the grounded collector, extending for a distance of 10 cm. A flat plate wrapped in aluminum foil was used as the collector to spin scaffolds with a random fibrous structure. The electrospun scaffolds were dried under vacuum at room temperature for 72 h.

The morphology of the electrospun scaffolds was observed using field emission scanning electron microscopy (Zeiss DSM 982, FESEM, Oberkochen, Germany) at 3 keV.

### 2.2. Cell Culture

MC3T3-E1 cells were cultured in alpha minimum essential medium (α-MEM) supplemented with 10% fetal bovine serum (FBS) (Corning, New York, NY, USA) and 1% pen-strep (Corning, New York, NY, USA). Cells were grown in a humidified atmosphere of 5% CO_2_ at 37 °C. The culture medium was changed every other day. An osteogenic medium, consisting of α-MEM plus 10 mM β-glycerol phosphate and 50 mg/mL-ascorbic acid (Sigma, St. Louis, MO, USA), was used for osteogenic differentiation of the cells.

Mouse bone marrow stromal cells (mBMSCs) were derived from 6–8-week-old mice. The study was conducted in accordance with relevant national legislation on the use of animals for research and the protocol was approved by the Ethics Committee of Zhejiang University Laboratory Animal Center. Briefly, mice were euthanized using CO_2_ asphyxiation. Femurs and tibias were dissected from surrounding tissues. Bone marrow was harvested by flushing bones with α-MEM medium containing 10% FBS using a 25-G needle. The cells were filtered through a 70 µm cell strainer and plated at a density of 1 × 10^8^ cells in 100 mm culture dish. Half of the culture medium was replaced by fresh medium to remove the non-adherent cells at day 4. The cells were allowed to grow for 7–8 days in α-MEM containing 10% FBS and then passaged. mBMSCs were used at passage 2 for all the experiments performed in this study.

### 2.3. ECM Decoration on Electrospun Nanofibers

PLLA nanofibrous (PLLA NF) was cut into 8.0 mm circular samples using a biopsy punch with a diameter of 8.0 mm. All the samples were then sterilized using ethylene oxide gas before cell culture. Sterile PLLA NF samples were glued to the bottom of a 24-well plate using pharma-grade Vaseline to prevent floating and soaked in cell culture medium for 12 h. After that, MC3T3-E1 cells were seeded on PLLA NF at 5 × 10^4^ cells/cm^2^. MC3T3-E1 cells were allowed to grow on PLLA NF for 2 weeks with osteogenic medium described previously. The culture was terminated after 2 weeks by washing the samples with PBS once and all the samples were fixed in 4% paraformaldehyde and buffered with PBS for 30 min. After that, all samples were collected in conical tubes and frozen in a −20 °C freezer overnight and then thawed in a 37 °C water bath. To remove all the cellular components of MC3T3-E1 cells, the cells were treated with 1% SDS (Life Technology, Waltham, MA, USA) in deionized water for 30 min (Figure S1). The decellularized samples were washed with PBS extensively to remove any detergent residue and cellular components. The obtained ECM decorated PLLA NF (ECM/PLLG NF) were kept in PBS at 4 °C for further use.

### 2.4. Cell Attachment and Morphology

mBMSCs were seeded on both PLLA NF and ECM/PLLA NF in a 24-well plate at a seeding density of 2 × 10^4^ cells/cm^2^ in 1.0 mL medium (*n* = 10). The cells were allowed to adhere to the test materials for 2 h. After that, all the cell culture medium was slowly aspirated, and all the samples were washed gently with PBS twice. Half of the samples were then fixed in 2.5% glutaraldehyde for 1 h. Then they were incubated in 0.1 mol/L sodium cacodylate buffer for 1 h. The specimens with fixed cells were dehydrated in graded ethanol series and critical point dried. All specimens were then sputter coated with gold–palladium. The cell morphology on different substrates was observed using FESEM (JEOL 6335F, Akishima, Japan) at an acceleration voltage of 2 keV. The other half samples were then washed by PBS three times to remove the unattached and loosely bound cells from material surfaces. CellTiter-Blue^®^ assay was used to measure the density of cells leſt on the samples. 0.5 mL fresh medium containing 10% CellTiter-Blue^®^ dye (Promega, Madison, WI, USA) was added to each well and incubated for 2 h. Aliquots of 200 µL from each well were transferred into a 96-well plate and read at (λ_ex_ = 570 nm/λ_em_ = 590 nm) using a fluorescence microplate reader (Bio-tek MQX, Winooski, VT, USA). The cell number on each sample was calculated based on a calibration curve prepared with predetermined cell numbers.

In another set of experiments, mBMSCs were seeded on both PLLA NF and ECM/PLLA NF in a 24-well plate at a seeding density of 2 × 10^4^ cells/cm^2^ in 1.0 mL medium (*n* = 5). The cells were allowed to grow on the specimens for 7 days. After that, cells were fixed by 4% paraformaldehyde in PBS for 20 min at room temperature. Cells were permeabilized in 0.5% Triton X-100 in PBS for 15 min. To visualize both actin and the nucleus, tetramethylrhodamine isothiocyanate (TRITC)-conjugated phalloidin and 0.5 μg/mL 4′,6-diamidino-2-phenylindole dihydro-chloride (DAPI) were added to the cells and incubated for 30 min. After extensive washing with PBS, the cells were imaged using a fluorescent microscope (Zeiss Axiovert 200M, Oberkochen, Germany) with filters appropriate for TRITC and DAPI.

### 2.5. Cell Metabolism

In another set of experiments, mBMSCs were seeded onto the scaffolds (PLLA NF and ECM/PLLA NF) at a density of 2 × 10^4^ cells/cm^2^ in 1.0 mL of medium (*n* = 8). At each predetermined time points (day 3, 7 and 14), each scaffold was carefully transferred into another 24-well. 0.5 mL 10% CellTilter Blue in α-MEM was added into each well and incubated at 37 °C for 2 h. Aliquots of 200 µL from each well were transferred into a 96-well plate and read at (λ_ex_ = 570 nm/λ_em_ = 590 nm) using a fluorescence microplate reader (Bio-tek MQX, Winooski, VT, USA). The cell number on each sample was calculated based on a calibration curve prepared with predetermined cell numbers.

Finally, the cell morphology on different substrates was examined using field emission scanning electron microscopy (FESEM, LEO/Zeiss DSM 982, Oberkochen, Germany). To assess the cell distribution and extracellular matrix deposition, mBMSCs were seeded and cultured on PLLA NF and ECM/PLLA NF for 14 days. After that, all the specimens were prepared as described earlier in this section and then subjected to FESEM observations.

### 2.6. ALP Activity

The osteogenic differentiation of mBMSCs was determined by measuring their alkaline phosphatase (ALP) activity. Briefly, after 3, 7 and 14 days of culture, all media were removed, and each specimen was washed with PBS twice. 200 µL lysis buffer containing 0.5% Triton X-100 was added into each well and incubated for 20 min. The cell lysis was then centrifuged at 3000× *g* at 4 °C for 5 min. Aliquots of supernatants were subjected to a total protein assay using a BCA assay kit (Thermo Scientific, Waltham, MA, USA). The ALP activity was measured by colorimetric assay with reagent mixture composed of 5 mM *p*-nitro-phenol phosphate disodium (*p*-NPP), 1 mM MgCl_2_, and 0.15 M 2-amino-2-methyl-1-propanol (AMP) (Sigma, St. Louis, MO, USA) together with an equal volume amount of nitrophenyl phosphate (10 mM). The optical density of the solution was measured at 405 nm using a microplate reader (Biotek, MQX200, Winooski, VT, USA). The ALP activity was expressed as total activity per microgram protein per sample.

### 2.7. Characterization of Mineralized Tissue Formation

Formation of mineralized tissue was assessed by Alizarin Red-S (ARS) staining at 14 days of osteogenic culture. The cultured specimens were washed with PBS and fixed in 10% (*v*/*v*) formaldehyde at RT for 30 min. The cells were then washed twice with excess dH2O prior to addition of 1 mL of 40 mM ARS (pH 4.1) per well for 30 min. After aspiration of the unincorporated ARS, the wells were washed four times with 4 mL dH2O while shaking for 10 min. The specimens were then imaged using a Cannon scanner (Tokyo, Japan). To quantify the amount of calcium deposited on each type of specimen, the mineral component was dissolved in 1.0 M HCl and the calcium concentration was measured as follows: 5 μL of sample solution was mixed with 195 μL of a 0.4 mM Arsenazo III prepared in 0.02 M Tris buffer (pH = 7.40). The amount of calcium quantitatively detected by measuring the absorbance of the 615 nm band on a microplate reader (Biotek, MQX200, Winooski, VT, USA) and compared it to a set of standards with known calcium concentrations.

### 2.8. Mineralization

To test the apatite formation capability on both PLLA NF and ECM/PLLA NF, the nanofibrous mats were incubated in modified simulated body fluid (mSBF) at 37 °C 7 days with continuous rotation. The mSBFs were prepared by adding the following reagents into deionized water in the order shown: 141 mM NaCl, 4.0 mM KCl, 0.5 mM MgSO_4_, 1.0 mM MgCl_2_, 4.2 mM NaHCO_3_, 20.0 mM HEPES, 5.0 mM CaCl_2_, and 2.0 mM KH_2_PO_4_. The pH of mSBF was then adjusted to 6.80 using HCl/NaOH. In particular, each nanofibrous mat was incubated in 50 mL mSBF in a conical tube to form the mineral coatings. The mSBFs were refreshed daily throughout the entire coating process in order to maintain consistent ion concentrations for mineral coating growth. After 7 days of incubation, the scaffolds were rinsed with deionized water and lyophilized. After that, the specimens were gently washed with de-ionized water and air dried. The formation of the apatite coating on the surface of titanium disks was then observed using FESEM (JEOL 6335F Akishima, Japan) at 5 kV.

### 2.9. Statistical Analysis

All quantitative data are given as mean ± standard deviation. The numerical results obtained in this study were subjected to a two-tailed Student’s *t*-test. The significance of the results was evaluated at a significance level of *p* < 0.05 based on the *p* value of comparison.

## 3. Results and Discussion

### 3.1. Generation and Characterization of ECM Decorated PLLA NF

Similar to decellularized tissues, cell-derived ECM provides a natural scaffold for cell attachment, proliferation and differentiation as it is a bioactive materials containing a series of fibrillary protein, matrix molecules, and embedded growth factors [36]. Therefore, the utility of cell-derived ECM for enhancing the performance of electrospun PLLA nanofibers is confirmed to be a feasible approach. Specifically, we used MC3T3-E1 cell line to generate ECM on PLLA NF surface, as its ECM contains osteogenic and osteoinductive factors accumulated during its differentiation.

**Figure 1:**
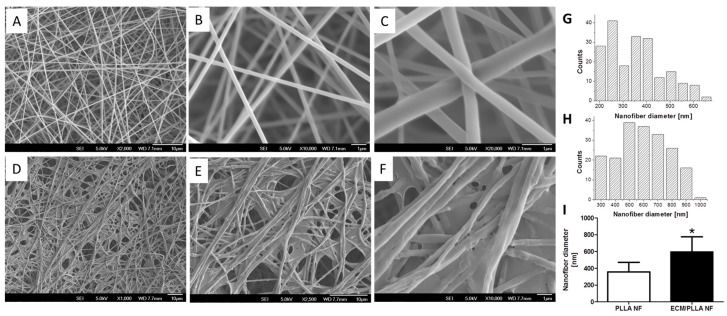
PLLA electrospun nanofibers before and after ECM decoration: (**A**) Electrospun PLLA Nanofibers, low mag; (**B**) Electrospun PLLA Nanofibers, medium mag; (**C**) Electrospun PLLA Nanofibers, high mag; (**D**) Electrospun PLLA Nanofibers decorated with ECM, low mag; (**E**) Electrospun PLLA Nanofibers decorated with ECM, medium mag ; (**F**) Electrospun PLLA Nanofibers decorated with ECM, high mag; (**G**) Histogram of PLLA NF diameter; (**H**) Histogram of ECM/PLLA NF diameter; (**I**) Average diameter comparison between PLLA and ECM/PLLA, * indicates significant difference (*p* < 0.05).

In order to obtain ECM decoration on PLLA NF surface, we employed a cell-based strategy to generate ECM coating on the nanofibrous PLLA surface. As shown in Figure 1A, nanofibers with uniform size were formed after electrospinnnig. Although fiber size variation can still be observed when zoomed in (Figure 1B,C,G,H), the average diameter of the nanofibers was around 350 nm based on image-based statistics (Figure 1I). After decorating with ECM, it was observed that a layer of ECM uniformly covered on the nanofiber while still preserving the nanoscale morphology of the electrospun fibers. Compared to nanofibers without ECM decoration, it was noticed that the average size of the nanofibers was slightly increased, which is probably due to the swelling of PLLA during long cell culture times.

### 3.2. Cell Attachment and Spread

**Figure 2:**
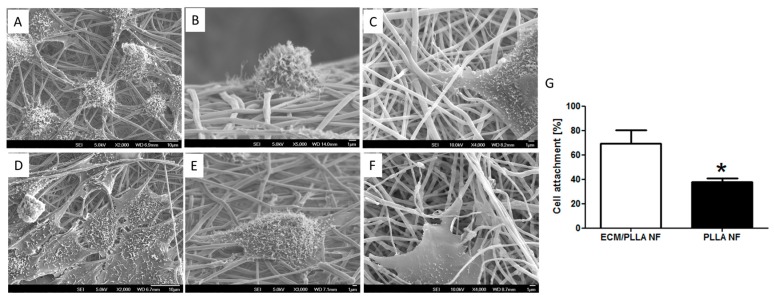
mBMSC adhered on PLLA electrospun nanofibers before and after ECM decoration: (**A**) Overview of mBMSCs on PLLA NF; (**B**) Single cell attached on PLLA NF; (**C**) Highlight of mBMSCs attached to PLLA nanofibers; (**D**) Overview of mBMSCs on ECM/PLLA NF; (**E**) Single cell attached on ECM/PLLA NF; (**F**) Highlight of mBMSCs attached to ECM/PLLA nanofibers. (**G**) mBMSC attachment on two scaffolds after 2 h; * indicates significant difference at *p* < 0.05.

Although polyester-based nanofibers fabricated by electrospinning have been widely used in tissue engineering and regenerative medicine [15], the cell attachment behaviors of these materials are still considered to be suboptimal due to their high hydrophobicity. We hypothesized that ECM decoration on electrospun nanofibers could significantly improve cell attachment by providing abundant adhesive components such as fibronectin, vitronectin, etc. on the materials’ surface [37,38]. This hypothesis was verified by showing a high cell attachment ratio on ECM/PLLA NF compared to PLLA NF (Figure 2G). The percentage of cells attached to ECM/PLLA NF was 30% more than for PLLA NF. The morphology of mBMSCs after being seeded on nanofibers is shown in Figure 2. Cells on PLLA NF exhibited a round morphology with limited filopodia extension (Figure 2A). The side view of individual cell showed that the cell only physically attached to the nanofiber, and no further interactions between the nanofibers and the cell were observed (Figure 2B,C). In contrast, rBMSCs grown on ECM/PLLA NF demonstrated a flat polygonal shape on the material surface, and most of them exhibited multipolar extensions. As shown in Figure 2E, cells on ECM/PLLA NF tend to stretch their filopodia along the nanofiber direction, indicating that the ECM coating on these fibers had remarkably enhanced their interactions with cell anchoring points. This feature of the ECM decorated nanofibers was clearly visible in high-magnification SEM images, which clearly showed that the cells spread following the orientation of the nanofibers. Therefore, ECM deposited by MC3T3-E1 cells can effectively improve cell attachment on electrospun nanofibers.

### 3.3. Cell Proliferation and Differentiation

**Figure 3:**
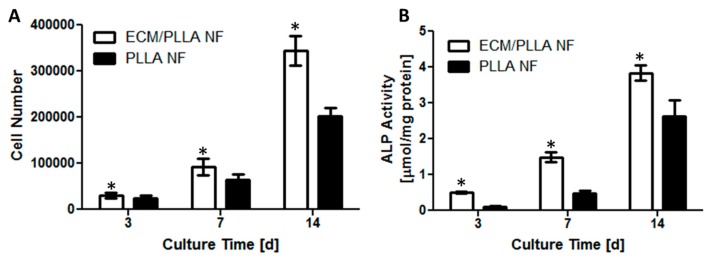
mBMSCs proliferation and osteogenic differentiation on PLLA NF and ECM/PLLA NF for 3, 7 and 14 days: (**A**) The cell number was significantly higher onECM/PLLA NF than on PLLA NF (*p* < 0.05) at all time points; (**B**) ALP activity normalized to total protein content of mBMSCs cultured on PLLA NF and ECM/PLLA NF for 3, 7 and 14 days. ALP activities were significantly higher on ECM/PLLA NF than on PLLA NF (*p* < 0.05) at all time points. * indicates significant differences.

To determine the effect of ECM decoration on mBMSCs viability, cell numbers on different treated PLLA nanofibers were recorded at day 3, 7 and 14. As shown in Figure 3A, cell numbers on both tested groups increased significantly as culture time extended. At day 3, the cell numbers on ECM/PLLA NF were slightly higher than those on PLLA NF, which might be due to the faster cell settlement on ECM-decorated nanofibers. The difference in cell numbers between ECM/PLLA NF and PLLA NF had become even more significant by day 7. At day 14, the cell numbers for ECM treated group were 70% higher than those for PLLA NF, indicating that the presence of ECM not only improved cell attachment at the initial stage, but also provided proliferative signal, stimulating the cells to grow faster.

ALP activity was evaluated as an early marker of osteogenic differentiation of mBMSCs cells. Although ALP activity in both groups was very low at day 3, it was noticed that ALP on ECM/PLLA NF was still slightly higher than PLLA NF group (Figure 3B). ALP activity on both tested groups increased gradually between 3 and 7 days, and there is a noticeable difference between ECM/PLLA NF and PLLA NF, suggesting that ECM presence on nanofiber surface could increase ALP activity, which may be further assigned to mineral deposition during MC3T3-E1 differentiation. A dramatic increase in ALP activity was observed during the second week of culture due to the switch of culture medium from basal medium to osteogenic medium. Although the ALP activity on PLLA NF surfaces increased by over 3 folds, the ALP activity on the ECM-treated surfaces were still much higher than the untreated group, which indicates that the ECM decoration approach used here is more favorable for osteogenic differentiation. Similar results have also been observed by other investigators in the context of bone tissue engineering. For instance, Sutha et al. showed in their study that embryonic stem cell-derived ECM possessed osteoinductive factors, which promoted ectopic bone formation in vivo [39]. Since MC3T3-E1 cell is an osteoblastic cell line which can differentiate into osteoblast, osteogenic factors can be secreted during their differentiation [40]. Thus, the enhanced osteogenic differentiation of mBMSCs on ECM/PLLA NF might be attributed to the osteogenic signals contained in MC3T3-E1 derived ECM.

### 3.4. Cell Morphology and Mineral Deposition

**Figure 4:**
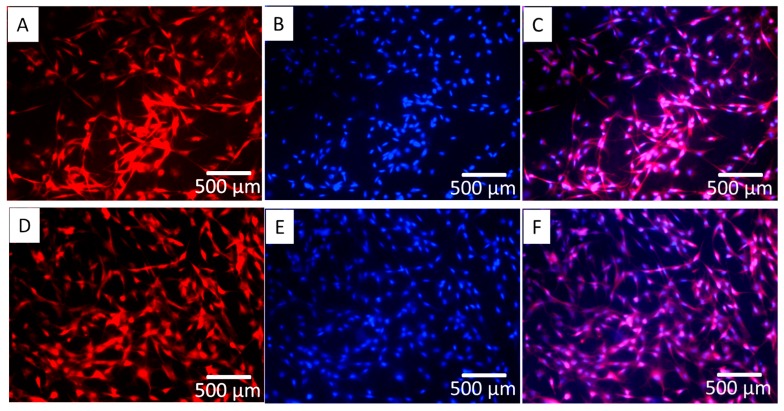
Immunostaining of actin, and nuclei showing mBMSC cells cultured on PLLA NF and ECM/PLLA NF, respectively: (**A**–**C**) actin staining, DAPI staining, and merged image of mBMSCs on PLLA NF at day 7; (**D**–**F**) actin staining, DAPI staining, and merged image of mBMSCs on ECM/PLLA NF at day 7; Red: actin; Blue: nuclei.

**Figure 5:**
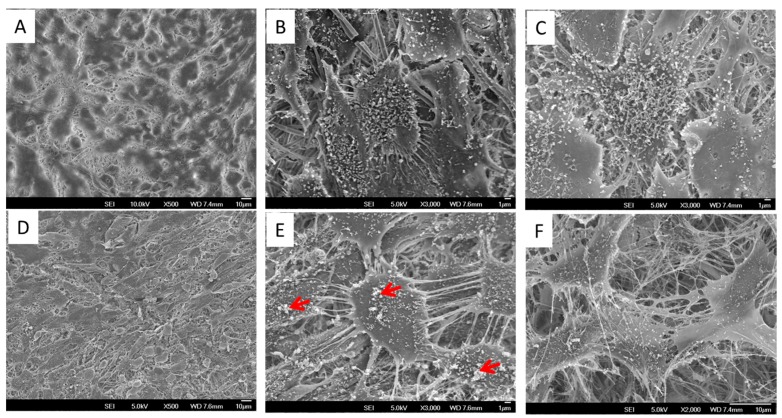
SEM micrographs showing mBMSCs morphologies cultured on PLLA and ECM/PLLA NF for 14 days. (**A**) PLLA (at low mag); (**B**) PLLA (at high mag); (**C**) PLLA (matrix deposition); and (**D**) ECM/PLLA (at low mag); (**E**) ECM/PLLA (at high mag); (**F**) ECM/PLLA (matrix deposition). It is shown that mBMSCs grown on ECM/PLLA NF developed multilayer structure and ECM deposition on this surface is more robust. Red arrow: mineralized nodule formation after culture.

Figure 4 shows the mBMSC cells growing on the two types of surface after 14 days of culture; however, cell coverage on ECM/PLLA NF appears to be more uniform compared to PLLA NF. Similar results were also observed in Figure 5 using SEM. Both surfaces were covered with a layer of cells. In particular, cells on PLLA NF exhibited a flatter morphology and tended to fully spread out. However, there are still small gaps between cells, and nanofibers were still exposed without cell layers covering them. Meanwhile, mBMSCs on ECM/PLLA NF grew tightly to each other and tended to form cell colonies (Figure 5D). Moreover, these cells demonstrated a spindle-shaped morphology, which is more similar to the morphology of osteoblasts. At a high magnification, although cells on PLLA NF showed a healthy morphology, the surface was only covered a single layer of cells. In comparison, ECM/PLLA NF surface was heavily covered by multiple layers of mBMSCs, which displayed numerous interactions between different cells (Figure 5B,E). Furthermore, we also found that new ECM deposition on ECM/PLLA NF was more abundant than PLLA NF. Mineralized nodules can be clearly seen on ECM/PLLA NF, whereas they are sparse on PLLA NF (Figure 5C,F), which implies that decoration of ECM secreted by MC3T3-E1 cells enhanced newly deposited ECM by BMSC cells and osteoblastic cells.

**Figure 6:**
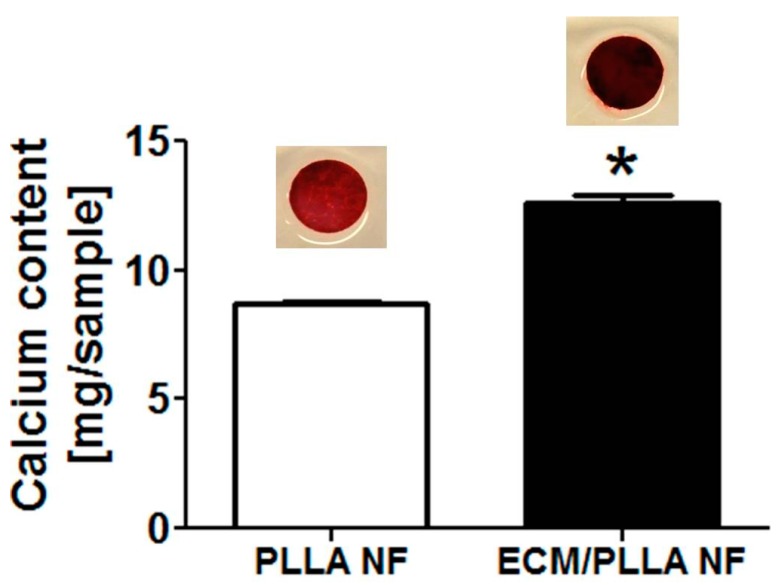
Calcium deposition on PLLA NF and ECM/PLLA NF after mBMSCs culture for 14 days: inserted images were Alizarin Red S staining of calcium. Calcium deposition were significantly higher on ECM/PLLA NF than on PLLA NF (*p* < 0.05) after 14 days. * indicates significant differences.

We also studied the mineral deposition in terms of calcium on both surfaces after two weeks. It was found that both groups were mineralized, showing a layer of calcium mineral on the surface (Figure 6). Nevertheless, it was found that calcium deposition on ECM/PLLA NF was about 12.59 mg/sample, which is much higher than that of PLLA NF (8.72 mg/sample); thus, decoration of MC3T3-E1 derived ECM substantially increased calcium deposition during mBMSC differentiation.

### 3.5. Biomineralization

**Figure 7:**
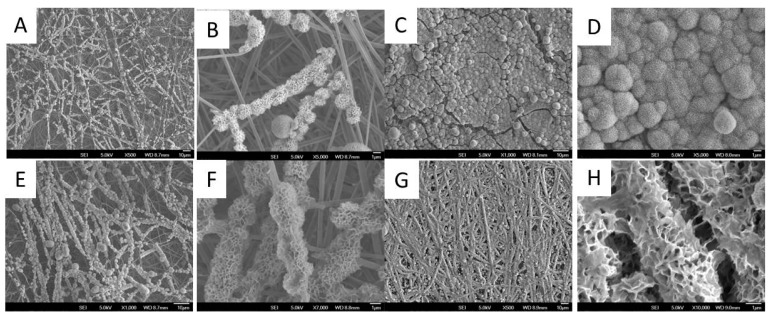
Apatite coating formation on two types of PLLA nanofibers with different treatment: (**A**) Apatite growth survey on PLLA NF at 24 h; (**B**) Apatite growth zoom-in on PLLA NF at 24 h; (**C**) Apatite growth survey on PLLA NF at 48 h; (**D**) Apatite growth zoom-in on PLLA NF at 48 h; (**E**) Apatite growth survey on ECM/PLLA NF at 24 h; (**F**) Apatite growth zoom-in on ECM/PLLA NF at 24 h; (**G**) Apatite growth survey on ECM/PLLA NF at 48 h; (**H**) Apatite growth zoom-in on ECM/PLLA NF at 48 h, after soaking in mSBF for different time periods.

Biomineralization capability is closely related to the bone-forming potential of biomaterials in vivo; therefore, it is commonly used to characterize the bioactivity of biomaterials [41]. To investigate the effect of ECM decoration on PLLA nanofiber biomineralization, both PLLA NF and ECM/PLLA NF were soaked in mSBF to evaluate their mineral-forming capability. As shown in Figure 7A, scattered mineral clusters were formed on PLLA NF after soaking in mSBF for 24 h. When zoomed in, it can be noticed that these minerals mainly grew along the fiber directions, instead of uniformly coating the surface (Figure 7A,B). On the other hand, mineral growth on ECM/PLLA NF showed a much faster trend. It was found that mineral coating had homogeneously formed along the nanofibers, which increased the diameter of the nanofibers from several-hundred nanometers to about 1 μm (Figure 7E,F). The localization of mineral coating on nanofibers indicates that mineral nucleation mainly occurred on the nanofibers, which may be due to the presence of MC3T3-E1-derived ECM. When we extended the biomineralization time to 48 h, it was found that a thick mineral coating covered the whole nanofiber surface, causing PLLA NF to lose its nanoscale features (Figure 7C,D). However, this situation did not occur with ECM/PLLA NF. Instead, the mineral coating continued to grow along the nanofiber direction, and preserved its nanoscale morphology (Figure 7G,H). The results from biomineralization indicate that ECM decoration can facilitate mineral deposition on PLLA nanofiber surface, which is beneficial for bone regeneration.

## 4. Conclusions

In conclusion, electrospun PLLA nanofibers were successfully decorated by ECM derived from osteoblastic cells by decellularization of MC3T3-E1 cells grown for two weeks. The results demonstrated that application of ECM on PLLA nanofibers enhanced mouse bone marrow stromal cell (mBMSC) adhesion, supported cell proliferation and promoted osteogenic differentiation of mBMSCs. Therefore, this study provided an alternative platform for directly translating the regenerative potential of ECM into clinical therapeutics and motivating the development of cell-derived ECM materials for broad regenerative medicine applications.

## Supplementary Materials

The following are available online at http://www.mdpi.com/2073-4360/10/3/272/s1.
