# Supplementary material for: ECM Decorated Electrospun Nanofiber for Improving Bone Tissue Regeneration
Source: Polymers (Basel). 2018 Mar 6;10(3):272. doi: 10.3390/polym10030272 (PMC6414933; doi:10.3390/polym10030272)
Supplement: Supplementary file 1 [file polymers-10-00272-s001.pdf]

polymers

Support Information

ECM Decorated Electrospun Nanofiber for Improving Bone Tissue Regeneration

Yong Fu [1,*], Lili Liu [2], Wendell Q. Sun[3], Ruoyu Cheng [2] and Wenguo Cui [2,4,*]

[1] Department of ENT and Head & Neck Surgery, The Children's Hospital Zhejiang University School of Medicine, 3333 Bingsheng Road, Zhejiang, Hangzhou 310051, China
[2] Orthopedic Institute, Soochow University, 708 Remin Road, Suzhou, Jiangsu 215006, China; 13295152709@163.com (L.L.); cry214657@126.com (R.C.)
[3] Institute of Bio-thermal Science and Technology, University of Shanghai for Science and Technology, Shanghai 200093, China; Wendell.q.sun@gmail.com (W. S)
[4] Shanghai Institute of Traumatology and Orthopaedics, Shanghai Key Laboratory for Prevention and Treatment of Bone and Joint Diseases, Ruijin Hospital, Shanghai Jiao Tong University School of Medicine, 197 Ruijin 2nd Road, Shanghai 200025, China
* Correspondence: 1307022@zju.edu.cn (Y.F.); wgcui80@hotmail.com (W.C.); Tel: +86-571-86670860.
Received: 25 January 2018; Accepted: 26 February 2018; Published: date

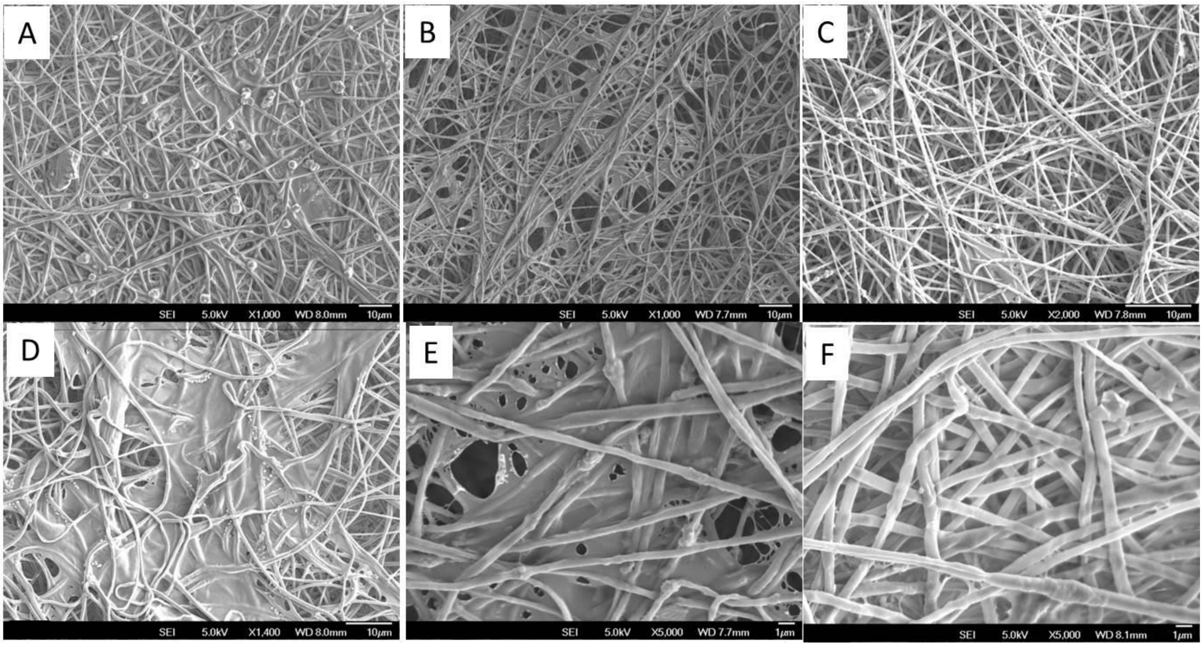

Figure S1. ECM/PLLA electrospun nanofibers after SDS treatment for different time period: A) 30 min, low mag B) 60 min, low mag C) Over night, low mag D) 30min, high mag ; E) 60 min, high mag, F) Over night, High mag.

MC3T3-E1 cell derived ECM was treated with 1%SDS for different time periods. When only treated for 30 min, the layer of ECM almost maintained its integrity, indicating that such short time treatment was not able to remove all the cellular components from the cells. **MC3T3-**E1 cell deposited ECM was then treated by SDS for 60 min, in turn we found that SDS already started to break down the structural component of the cells, but the ECM layer still covered the majority of the surface. When SDS treatment time was extended to overnight, it was observed that the whole layer of ECM was destroyed and no visible membrane can be seen under SEM. In summary, 60 min of decellularization with 1% SDS was chosen as the optimal condition for ECM preparation.

© 2018 by the authors. Submitted for possible open access publication under the terms and conditions of the Creative Commons Attribution (CC BY) license (http://creativecommons.org/licenses/by/4.0/).
